# Frequency of Left Ventricular Hypertrophy Among Patients on Maintenance Hemodialysis by Voltage Criteria and Its Relationship with Biophysical-Chemical Parameters

**DOI:** 10.7759/cureus.7426

**Published:** 2020-03-26

**Authors:** Alvina Zanib, Shahid Anwar, Khurram Saleem, Hafiz Muhammad Wasif Khan, Sana Zafar

**Affiliations:** 1 Internal Medicine, Fatima Jinnah Medical University, Lahore, PAK; 2 Nephrology, Fatima Jinnah Medical University, Lahore, PAK; 3 Internal Medicine, University College of Medicine, University of Lahore, Lahore, PAK; 4 Internal Medicine, Fatima Jinnah Medical University, Sir Ganga Ram Hospital, Lahore, PAK

**Keywords:** hemodialysis, electrocardiogram (ecg), end stage renal disease (esrd), left ventricular hypertrophy (lvh)

## Abstract

Background

Among the dialysis population, left ventricular hypertrophy (LVH) is becoming a major cause of cardiovascular death, mainly due to myocardial infarction, heart failure, and arrhythmias. Electrocardiography (ECG) is a cheap and easily available test to detect the presence of left ventricular hypertrophy. The basic purpose of this study was to assess the frequency of left ventricular hypertrophy among the maintenance hemodialysis patients by applying different voltage criteria for the diagnosis of LVH and its relationship with various biophysical and biochemical parameters.

Methods

A total of 68 patients of end-stage renal disease (ESRD) were included in the study who were on maintenance hemodialysis at the dialysis center of Sughra Shafi Hospital. Baseline characteristics were recorded from the patients' data. Blood samples were drawn and electrocardiographs were taken, both before and after hemodialysis.

Results

Results showed variability in the detection of left ventricular hypertrophy in the pre- and post-dialysis period, as it was positive for 45%, 21%, and 17% in the pre-dialysis period versus 40%, 32%, and 25% in the post-dialysis period, when the Framingham, Sokolow-Lyon, and Cornell criteria were applied, respectively. The study showed a significant relationship between left ventricular hypertrophy with a high body mass index (BMI), hypertension, and pre- and post-dialysis hypomagnesemia (P <0.05). A significant association was also seen with low serum albumin levels over the past year.

Conclusion

According to this study, almost half of the dialysis patients were having left ventricle hypertrophy when Framingham criteria were applied. Good control of factors that are significantly associated with the occurrence of left ventricular hypertrophy can reduce morbidity and mortality among dialysis patients secondary to cardiovascular events. In this study, these factors included hypertension, hypomagnesemia, hypoalbuminemia, and high BMI.

## Introduction

Cardiac diseases, including left ventricular hypertrophy (LVH), are the leading cause of death among the dialysis population. The overall mortality from cardiovascular diseases remains 43%-52% in the end-stage renal disease (ESRD) population. Left ventricular hypertrophy is an important factor that portrays the systolic-diastolic dynamics of the left heart in the hemodialysis population. About 80% of the dialysis population has various forms of cardiovascular involvement; this entity being part of the spectrum of cardiorenal syndromes. In chronicity, it has been categorized as cardiorenal syndrome type IV [1-4].

A previous study conducted on a large scale documented that 25 cardiovascular deaths/1000 patients per year were associated with LVH. This was a greater absolute risk than that associated with diabetes mellitus, smoking, and hypertension. Reducing the progression of LVH in ESRD patients is associated with a reduction in all-cause and cardiovascular mortality [5-6].

LVH becomes increasingly common as the glomerular filtration rate (GFR) falls and the duration of maintenance dialysis increases. Almost 75% of patients were found to have LVH proven on echocardiography at the start of dialysis [7]. Both types of hypertrophies, concentric or eccentric, may be found secondary to pressure and volume overload, respectively [8].

Electrocardiography (ECG) has the benefits of being an easy-to-perform, non-invasive, widely available, cheap, and easily reproducible test. The different voltage criteria for diagnosing LVH have high specificities (>90%) but lower sensitivities 20%-60% [9-10]. However, data on the diagnostic value of ECG in hemodialysis patients is quite limited [11-12].

The purpose of this study was to assess the frequency of LVH among hemodialysis patients using three different voltage criteria. Meanwhile, we compared changes in hypertrophy with pre- and post-dialysis ECG. Furthermore, we tried to establish an association between LVH by Framingham criteria and other variables, e.g. age, gender, duration of renal replacement therapy, ultra-filtrate volume, body mass index (BMI), lipid profile, pre- and post-blood pressure, urea, creatinine, and electrolytes. In addition, we compared LVH by Framingham criteria with values of mean hemoglobin, calcium, phosphate, albumin, uric acid, urea, and creatinine over the past one year.

## Materials and methods

In this study, 70 patients on thrice-weekly maintenance hemodialysis at Sughra Shafi Hospital, Narowal, were enrolled during July 2019. All males and females above 18 years were included in this study. Patients having atrial fibrillation, Wolf Parkinson White syndrome, bundle branch block, or pacemaker were excluded.

After getting ethical approval and informed written consent, data were recorded from all patients regarding their age, gender, cause of renal disease, time since on hemodialysis, and comorbidities like ischemic heart disease, diabetes mellitus, and hypertension. Systolic and diastolic arterial pressure along with blood biochemistry, including serum magnesium, calcium, albumin, urea, creatinine, potassium, sodium, bicarbonate, and blood sugar level, were taken pre- and post-dialysis. The serum cholesterol profile, including high-density lipoprotein (HDL), low-density lipoprotein (LDL), and triglycerides, was also collected before the start of dialysis.

A 12-lead electrocardiogram (ECG) was taken in all the patients before and after a hemodialysis session and interpreted for the presence or absence of left ventricular hypertrophy on the basis of different voltage criteria. LVH was labeled by Sokolow-Lyon criteria, i.e, if the sum of S wave in V1 and R wave in V5 or V6 ≥3.5 mV (35 mm). While Cornell voltage criteria for men involve S in V3 plus R in aVL >2.8 mV (28 mm) while for women, it was S in V3 + R in aVL >2.0 mV (20 mm). Framingham criteria involved anyone of the following: R in aVL > 1.1 mV, or R in V5 or V6 > 2.5 mV, or S > 2.5 mV in right precordial leads, or the sum of SV1 or SV2 plus RV5 or RV6 > 3.5 mV, or the sum of RI and SIII >2.5 mV.

Statistical analysis was done through SPSS version 21.0 (IBM Corp, Armonk, NY). All categorical variables were presented in frequencies and percentage form. All quantitative variables were described in mean and standard deviation form. The paired t-test was used for paired samples (pre- and post-dialysis) to determine statistical significance. Significant differences in proportions were assessed by the chi-square test. P-values <0.05 were considered significant.

## Results

A total of 68 dialysis patients out of 70 were included in this study. Two patients were excluded because of atrial fibrillation. The mean age was 47 (SD 13), the male-to-female ratio was 1.9:1, and the average BMI was 24.6. The mean duration of renal replacement therapy was four years. Baseline characteristics have been shown in Table 1.

**Table 1:** Baseline characteristics of patients

Characteristics	Frequency (percentage)
Male	44 (64)
Female	24 (36)
Ischemic heart disease	16 (23.5)
Hypertension	61 (89.7)
Diabetics	32 (47)

The frequency of left ventricular hypertrophy by different voltage criteria has been given in Table 2.

**Table 2:** Frequency of left ventricular hypertrophy by different criteria

	Pre-dialysis Frequency (Percentage)		Post-dialysis frequency (percentage)	
	Positive	Negative	Positive	Negative
Sokolow-Lyon	21(30)	49(70)	32(45.7)	38(54.3)
Cornell	17(24.3)	53(75.7)	25 (35.7)	45(64.3)
Framingham	32(45.7)	38(54.3)	40(57.1)	30(42.9)

The mean values of biochemical parameters before and after hemodialysis have been given in Table 3.

**Table 3:** Mean values of biochemical parameters before and after hemodialysis

Parameter	Before hemodialysis (Mean ± SD)	After hemodialysis (Mean ± SD)
Serum sodium (meq/L)	141.4 (6.5)	142.7 (5.9)
Serum potassium (meq/L)	5.0 (0.9)	3.6 (1.0)
Serum magnesium (meq/L)	2.1 (0.6)	1.6 (0.4)
Serum calcium (mg/dl)	8.0 (1.0)	8.6 (0.6)
Serum bicarbonate (mg/dl)	12.6 (4.6)	17.7 (3.5)
Serum urea (mg/dl)	108.9 (29.6)	37.8 (15.4)
Serum creatinine (mg/dl)	8.2 (2.4)	3.3 (1.2)
Blood sugar level (mg/dl)	157.3 (82.1)	139.7 (51.6)
Serum albumin (g/dl)	3.9 (0.43)	4.3 (0.5)

A significant association was found among LVH by Framingham criteria on ECG and high BMI (0.01 and 0.007), history of hypertension (0.008 and 0.01) and pre- and post-dialysis serum magnesium levels (0.09 and 0.035). LVH was found more predominantly in male patients but with no significant relationship. Also, a significant relationship was observed among LVH by Framingham criteria on ECG and mean serum albumin level over the last year (P<0.02 and 0.04 pre- and post-dialysis ECG, respectively) but not with mean hemoglobin, calcium, phosphate, uric acid, urea, and creatinine level over the year. No significant relationship was found among LVH by Framingham criteria on ECG and age, duration of renal replacement therapy, history of DM, and IHD. Similarly, there was no significant relationship with pre- and post-dialysis serum albumin, calcium, sodium, potassium, bicarbonate levels, serum triglycerides, total cholesterol, HDL, and LDL levels.

## Discussion

According to this study, 45% of patients have LVH by Framingham criteria on ECG, 21% of patients have LVH by Sokolow-Lyon criteria, and 17% of patients are found to have LVH by Cornell criteria of LVH on ECG. Therefore, these results favor the increased sensitivity of Framingham criteria as proven in previous studies [13]. Meanwhile, the percentage of patients having LVH increased in the post-dialysis period to a value of 32% and 25% (from 21% and 17% pre-dialysis) by Sokolow-Lyon and Cornell criteria, respectively, and decreased to 40% (from the 45% pre-dialysis value) by Framingham criteria.

A significant association of LVH by Framingham criteria was found with patients having high BMI and a history of hypertension as seen in previous studies [14-16]. Pre- and post-dialysis hypomagnesemia was also related significantly to the presence of LVH detected on ECG by Framingham criteria. However, there was no relationship with pre- and post-dialysis serum albumin, calcium, sodium, potassium, and bicarbonate levels with LVH. Similarly, a significant association has been observed with mean serum albumin level over the past one year, but no relationship was found with mean hemoglobin, calcium, phosphate, uric acid, and urea and creatinine levels over the past one year. However, recent studies showed that LVH is related to low hemoglobin and high phosphate levels [17-19]. They also documented a reduction in LVH with a reduction in serum phosphate levels [19].

As seen in older studies, this study showed that LVH is more predominant in male patients as compared to females but with no significant relationship. This can be explained with low voltage QRS complexes in chest leads secondary to the presence of breast tissue in females. No significant relationship was seen with a history of diabetes mellitus (DM), ischemic heart disease (IHD), smoking, and serum triglycerides, total cholesterol, HDL, and LDL levels.

The main strength of this study was that all of these patients were under ideal conditions for maintenance hemodialysis, four-hour sessions thrice weekly. While the major limitation in this study was the small sample size. These results are reflecting just 70 hemodialysis patients. So, in the author’s opinion, future studies focusing on a large number of hemodialysis patients need to be conducted to get more accurate results. The authors are also planning to follow these 70 patients in the future, to assess mortality.

## Conclusions

In conclusion, almost half of the dialysis patients from this study were having LVH by Framingham criteria, showing such a high prevalence of LVH in the dialysis population. In addition, according to this study, many factors (e.g., high BMI, a history of hypertension, serum magnesium, and serum albumin) are found to be associated with the increased occurrence of left ventricular hypertrophy among dialysis patients. So taking good control of these factors and early detection of left ventricular hypertrophy can help in the reduction of high mortality among dialysis patients secondary to cardiovascular events, which is a leading cause of death in the dialysis population.
